# Nerve block reduces the incidence of 3-year postoperative mortality: a retrospective cohort study

**DOI:** 10.3389/fsurg.2024.1284892

**Published:** 2024-02-01

**Authors:** Lu Li, Chen xi Li, Hui Zhang, Jiaqiang Zhang

**Affiliations:** Department of Anesthesiology and Perioperative Medicine, People’s Hospital of Zhengzhou University, Henan Provincial People’s Hospital, Zhengzhou, Henan, China

**Keywords:** hip replacement, postoperative mortality, nerve block, agedness, retrospective cohort study

## Abstract

**Purpose:**

A retrospective cohort study was performed to determine the effect of nerve block on the incidence of postoperative mortality in patients with hip replacement.

**Methods:**

According to the inclusion and exclusion criteria, patients who were undergoing hip replacement for the first time under general or intraspinal anesthesia, classified as ASA class I–IV, and aged ≥65 years were selected. We collected the general data, past medical history, preoperative laboratory test results, perioperative fluid intake and outflow data, perioperative anesthesia and related drug data, postoperative laboratory results, and correlation time index. Patients with preoperative combined nerve block were included in the N group, and those without combined nerve block were included in the NN group. The patients were followed up via telephone call to assess survival outcomes at 3 years after surgery. Propensity score matching and uni- and multivariate analyses were performed to determine the influence of nerve block and other related factors on postoperative mortality.

**Results:**

A total of 743 patients were included in this study, including 262 in the N group and 481 in the NN group. Two hundred five patients in both groups remained after propensity score matching. Main result: Preoperative nerve block was associated with reduced mortality three years after surgery.

**Conclusion:**

Nerve block reduces the incidence of 3-year postoperative mortality, and composite nerve block with general anesthesia and neuraxial anesthesia is worthy of promotion.

## Introduction

Hip fractures occur in 1.5 million people annually worldwide, with only 26% occurring in Asia. The 1-year mortality rate after sustaining a fracture can be as high as 28% (1). At present, surgery is the most effective treatment for hip fracture, and elderly patients have reduced physiological function and often have hypertension, diabetes mellitus, coronary heart disease and other basic diseases, so the mortality rate of hip fracture is high. Prior studies have documented an association between pain and mortality (2), particularly for severe pain (3), multisite pain (4), severe chronic pain (5), widespread chronic pain (6, 7), or extreme pain interference (8).

With the development of anesthesia technology, ultrasound-guided nerve block has been more widely used in the clinic, and preoperative nerve block can more effectively relieve pain in patients with hip fracture than traditional opioids despite their rapid onset of action and few systemic adverse effects, thus making such a method worthy of substantial promotion. Thus, whether nerve block affects the incidence of postoperative mortality in postsurgical patients and the factors that affect the risk of postoperative mortality in such patients must be determined through further exploration. In this study, we retrospectively analyzed the perioperative clinical data, including patient factors, anesthesia-related factors and perioperative drug-related factors, of patients to explore the effect of nerve block as well as the incidence of postoperative mortality in hip arthroplasty patients.

## Materials and methods

This study was approved by the hospital Ethics Committee [Ethics No. (2019) No. (46)].

Corresponding clinical data were extracted using Lex clinical data application 3.2 from the hospital health information system (HIS) and the Madiston surgical anesthesia system to identify cases meeting the inclusion and exclusion criteria. Inclusion criteria: patients who were undergoing hip replacement for the first time under general or intraspinal anesthesia, classified as ASA class I–IV, aged ≥65 years, and with hip fracture (intertrochanteric fracture, femoral neck and hip fracture were included in the diagnosis). Exclusion criteria: patients with a history of malignancy or a history of multiple postoperative surgeries, patients who died accidentally after surgery due to trauma, car accident, etc., or who were lost to telephone follow-up. The patients who underwent hip replacement in our hospital from May 2016 to May 2019 were considered the study subjects. According to the case data, the patients with preoperative combined “bow tie sign” iliofascial nerve block were the N group, and those without preoperative nerve block were the NN group. The follow-up duration was ≥3 years.

Patient data: General data, including name, sex, age, weight, contact info for the patient and family members, weight, smoking history, alcohol abuse history, and allergy history, were collected. Previous histories of underlying diseases and surgical trauma, such as hypertension, diabetes mellitus, chronic obstructive pulmonary disease (COPD), coronary heart disease, myocardial infarction, valvular heart disease, cerebral hemorrhage, cerebral infarction, preoperative pulmonary infection, and asthma, were recorded. The patient's preoperative laboratory tests included the following: hemoglobin, white blood cells, serum creatinine, alanine aminotransferase, aspartate aminotransferase, albumin and BNP. Data on preoperative waiting time, ASA grade, blood loss volume, blood transfusion, colloid fluid use, and crystalloid fluid use were also collected. The following anesthetics and related medications were applied and administered intraoperatively: propofol, etomidate, cisatracurium, rocuronium bromide, tranexamic acid, sufentanil, remifentanil, dexmedetomidine, vasoactive drugs (methoxamine, phenylephrine, norepinephrine, dopamine, epinephrine), and atropine. The duration of surgery, interval from extubation (time from end of surgery until laryngeal mask extraction), and length of postanesthesia care unit (PACU) stay were monitored. The following analgesics and medications were used postoperatively: dexmedetomidine and anticoagulant drugs (aspirin, clopidogrel, warfarin, heparin). The following laboratory results obtained one day after surgery were collected postoperatively: hemoglobin, white blood cells, serum creatinine, alanine aminotransferase, aspartate aminotransferase, albumin and BNP, postoperative hospital days, and total hospital days.

Patients were followed up 3 years postoperatively by telephone by a dedicated person to assess the length of their survival, and the time to death was determined if the patient had died during the follow-up period.

Indicators with more than 35% missing indicator rows and more than 55% missing columns were excluded, and 43 variables were finally included. Random forest imputation was performed employing the R mice package. Data were tested for a normal distribution, those that conformed to a normal distribution were expressed as the mean (median) ± standard deviation (SD), and those that did not conform to a normal distribution were expressed as the mean (mean) and quantile (IQR). Univariate logistic regression was performed on a population-wide basis for all indicators; see the univariate table. Variables with a univariate indicator *P* < 0.2 were included in the multivariate model. Building a multivariate model: Seven indicators were determined by forward and backward stepwise regression, and a multivariate parameter table was exported, containing indicators or values (see the multivariate table). The two groups were matched according to age, sex, blood loss, preoperative waiting days, operation duration and preoperative laboratory test results, and univariate and multivariate tables were built.

## Result

According to the inclusion and exclusion criteria, after excluding patients who were lost to follow-up and those with more missing data, a total of 743 patients were included in this study, including 262 in the N group and 481 in the NN group. Two hundred five patients in both groups remained after propensity score matching. Before matching, there were 36 deaths, and after matching, the 3-year mortality rate was 13.7% (110 deaths) in the N group and 22. 9% in the NN group. After matching, there were 32 deaths, and the 3-year mortality rate was 15.6% (44 deaths) in the N group and 21.5% in the NN group. The results collected before and after matching are shown below:

The results of the univariate analysis showed that the 3-year mortality rate was lower in the N group than in the NN group before matching, but the difference was not statistically significant after matching. There was no statistically significant difference in the proportion of patients with preoperative comorbid hypertension, intraoperative vasoactive drug use, blood loss, colloid fluid infusion, postoperative analgesia, who received additional postoperative pain medications, postoperative anticoagulation therapy or had a long duration of surgery and there was also no significant difference in their preoperative activated partial thromboplastin time or preoperative hemoglobin level before matching. There were statistically significant differences in anesthesia methods after propensity score matching. See Table 1.

**Table 1:** Comparison of the general condition of patients and various perioperative indexes between the two groups.

Factor	Before matching			After matching		
	Group NN (*n* = 481)	Group N (*n* = 262)	*P-*value	Group NN (*n* = 481)	Group N (*n* = 262)	*P-*value
Mortality (%)	110 (22.9)	36 (13.7)	0.004	44 (21.5)	32 (15.6)	0.162
Sex (%)			0.208			0.836
Male	160 (33.3)	100 (38.2)		70 (34.1)	73 (35.6)	
Female	321 (66.7)	162 (61.8)		135 (65.9)	132 (64.4)	
Mean age	75.34 (9.11)	75.58 (9.53)	0.735	75.22	76.58	0.141
Age stratification			0.642			0.146
(65, 69]	153 (31.8)	84 (32.1)		72 (35.1)	56 (27.3)	
(69, 79]	146 (30.4)	76 (29.0)		51 (24.9)	61 (29.8)	
(79, 89]	158 (32.8)	83 (31.7)		73 (35.6)	71 (34.6)	
(89, 103]	24 (5.0)	19 (7.3)		9 (4.4)	17 (8.3)	
ASA classification (%)			0.539			0.09
II	222 (46.2)	114 (43.5)		97 (47.3)	79 (38.5)	
III	259 (53.8)	148 (56.5)		108 (52.7)	126 (61.5)	
Classification (%)						
Hypertension	134 (27.9)	113 (43.1)	<0.001	77 (37.6)	80 (39.0)	0.839
Diabetes	66 (13.7)	42 (16.0)	0.457	38 (18.5)	31 (15.1)	0.428
Coronary heart disease	76 (15.8)	53 (20.2)	0.155	39 (19.0)	40 (19.5)	1
Valvulae heart disease	228 (47.4)	108 (41.2)	0.124	93 (45.4)	84 (41.0)	0.425
Cerebrovascular disease	74 (15.4)	57 (21.8)	0.038	37 (18.0)	47 (22.9)	0.271
Method of anesthesia (%)			0.07			0.004
Intraspinal anesthesia	213 (44.3)	135 (51.5)		85 (41.5)	115 (56.1)	
General anesthesia	268 (55.7)	127 (48.5)		120 (58.5)	90 (43.9)	
Intraoperative drug use (%)						
Vasoactive drug (%)	225 (46.8)	206 (78.6)	<0.001	89 (43.4)	94 (45.9)	0.691
Dexmedetomidine (%)	97 (20.2)	68 (26.0)	0.085	45 (22.0)	54 (26.3)	0.356
Tranexamic acid (%)	63 (13.1)	45 (17.2)	0.162	31 (15.1)	26 (12.7)	0.568
Blood loss volume (ml)			0.004			0.992
(0–50]	69 (14.3)	43 (16.4)		33 (16.1)	31 (15.1)	
(50, 100]	89 (18.5)	75 (28.6)		54 (26.3)	52 (25.4)	
(100, 200]	144 (29.9)	74 (28.2)		61 (29.8)	65 (31.7)	
(200, 300]	83 (17.3)	27 (10.3)		21 (10.2)	22 (10.7)	
(300, +]	96 (20.0)	43 (16.4)		36 (17.6)	35 (17.1)	
Colloidal fluid (%)	225 (46.8)	206 (78.6)	<0.001	146 (71.2)	152 (74.1)	0.579
Analgesia for 3 days after surgery (%)	275 (57.2)	204 (77.9)	<0.001	154 (75.1)	156 (76.1)	0.908
Postoperative dexmedetomidine (%)	97 (20.2)	68 (26.0)	0.085	19 (9.3)	30 (14.6)	0.128
Add pain medication after surgery (%)	371 (77.1)	247 (94.3)	<0.001	187 (91.2)	190 (92.7)	0.717
Postoperative anticoagulation (%)	318 (66.1)	238 (90.8)	<0.001	186 (90.7)	182 (88.8)	0.625
[1, 2]	87 (18.1)	60 (22.9)		43 (21.0)	41 (20.0)	
3	98 (20.4)	53 (20.2)		42 (20.5)	47 (22.9)	
4	70 (14.6)	51 (19.5)		41 (20.0)	34 (16.6)	
[5, 7]	148 (30.8)	72 (27.5)		60 (29.3)	60 (29.3)	
7+	78 (16.2)	26 (9.9)		19 (9.3)	23 (11.2)	
Operation duration [media (IQR)]	1.75 [1.42, 2.25]	1.95 [1.53, 2.42]	<0.001	2.08 (0.90)	2.03 (0.80)	0.578
Preoperative laboratory						
Glutamic-pyruvic transaminase [median (IQR)]	16.00 [12.00, 23.00]	15.00 [12.00, 20.00]	0.052	20.22 (15.74)	17.99 (11.38)	0.1
Glutamic-oxalacetic transaminase [median (IQR)]	20.00 [16.00, 25.00]	19.00 [16.00, 23.00]	0.394	21.82 (9.68)	22.39 (14.60)	0.641
Albumin [mean (SD)]	35.13 (4.64)	36.38 (4.30)	<0.001	35.76 (4.57)	35.62 (4.21)	0.744
Prothrombin time [median (IQR)]	12.60 [11.80, 13.40]	12.60 [11.90, 13.60]	0.153	12.74 (1.64)	12.93 (1.38)	0.192
Activated partial thromboplastin time [median (IQR)]	35.30 [31.90, 38.22]	33.03 [29.60, 36.70]	<0.001	34.75 (7.93)	34.14 (5.10)	0.357
Thrombin time [median (IQR)]	17.20 [15.80, 18.30]	17.10 [16.00, 18.30]	0.897	17.19 (4.78)	16.94 (1.92)	0.495
Hemoglobin [mean (SD)]	111.39 (20.31)	117.48 (19.96)	<0.001	115.80 (19.71)	114.61 (19.22)	0.537
Hemameba [median (IQR)]	7.04 [5.65, 8.78]	6.60 [5.53, 7.89]	0.005	7.14 (2.75)	7.09 (2.36)	0.819
Postoperative laboratory examination						
Hemoglobin [mean (SD)]	98.95 (15.06)	101.40 (14.85)	0.034	99.94 (15.16)	100.73 (15.21)	0.599
Hemameba [mean (SD)]	9.70 (3.03)	9.49 (2.74)	0.347	9.72 (2.99)	9.50 (2.68)	0.433
Creatinine [median (IQR)]	57.00 [49.00, 69.00]	57.00 [49.00, 72.00]	0.972	65.81 (44.93)	66.88 (47.73)	0.816
Length of stay [median (IQR)]	14.00 [11.00, 19.00]	12.50 [10.00, 16.00]	0.001	14.35 (6.36)	14.38 (7.17)	0.971
Postoperative hospital stay [median (IQR)]	9.00 [7.00, 13.00]	8.00 [7.00, 11.00]	0.034	9.94 (5.48)	9.77 (5.44)	0.745
Crystalloid solution (%)			<0.001			0.993
0–500 ml	243 (50.5)	75 (28.6)		64 (31.2)	66 (32.2)	
(500, 1,000] ml	90 (18.7)	75 (28.6)		57 (27.8)	57 (27.8)	
(1,000, 1,500] ml	87 (18.1)	73 (27.9)		48 (23.4)	48 (23.4)	
(1,500, +] ml	61 (12.7)	39 (14.9)		36 (17.6)	34 (16.6)	

Logistic regression analysis showed that preoperative anticoagulation before matching, 0–500 ml fluid infusion, and preoperative prothrombin time were risk factors for 3-year mortality in patients. However, the combination of nerve block, general anesthesia, and postoperative analgesia reduced the risk of mortality at 3 years postoperatively. After propensity score matching, preoperative prothrombin time was a risk factor for 3-year mortality, and nerve block was associated with reduced mortality three years after surgery. See Table 2.

**Table 2:** Results of multifactor analysis before and after matching.

Factor	Before matching			After matching		
	OR	95% CI	*P*-value	OR	95% CI	*P*-value
Nerve block	0.567	0.351–0.901	0.018	0.558	0.319–0.963	0.038
General anesthesia	0.473	0.28–0.785	0.004	0.590	0.274–1.235	0.168
Postoperative analgesia	0.467	0.274–0.797	0.005	0.966	0.352–2.889	0.948
Postoperative anticoagulation	2.428	1.446–4.182	0.001	0.790	0.333–2.019	0.604
Preoperative hemoglobin	0.99	0.979–1.001	0.068	0.985	0.971–1	0.055
Preoperative albumin	0.945	0.9–0.991	0.021	0.949	0.89–1.012	0.107
Crystalloid solution (0–500]	2.734	1.345–5.91	0.007	2.011	0.793–5.413	0.152
Crystalloid solution (500, 1,000]	1.595	0.739–3.592	0.244	1.592	0.632–4.222	0.333
Crystalloid solution (1,500, +]	1.765	0.705–4.413	0.221	2.004	0.726–5.667	0.181
Tranexamic acid during surgery	0.491	0.218–0.995	0.064	0.778	0.296–1.811	0.581
Preoperative prothrombin time	1.277	1.119–1.465	0	1.335	1.12–1.619	0.062
Preoperative waiting days 3 days	0.624	0.311–1.229	0.176	0.657	0.268–1.563	0.346
Preoperative waiting days 4 days	1.157	0.596–2.241	0.665	1.388	0.624–3.11	0.421
Preoperative waiting days [5, 7] days	0.855	0.477–1.545	0.601	0.796	0.363–1.747	0.566
Preoperative waiting days 7+ days	1.626	0.835–3.172	0.152	1.472	0.534–3.904	0.442

## Discussion

Hip fractures cause moderate to severe pain, and pain can lead to increased blood pressure, tachycardia, and arrhythmias in patients. For patients with coronary heart disease, pain can lead to myocardial ischemia or even myocardial infarction. Pain can lead to enhanced platelet adhesion function and reduced fibrinolytic capacity, which put the body in a hypercoagulable state, thus increasing the risk of cardiovascular thrombosis in patients. Regional and widespread pain leads to long-term reduced levels of physical exercise that ultimately lead to an increased risk of cancer and cardiovascular mortality. Therefore, timely management of pain in elderly patients with hip fracture is an important component to ensure the perioperative safety of patients.

With the development of anesthesia technology, ultrasound-guided nerve block technology is being increasingly applied in the clinic, and iliofemoral nerve block is being increasingly applied as part of perioperative pain management for hip fracture patients because of its simple operation, high safety, and reliable results (9). After the patient enters the operating room, a bow-tie sign iliofascial nerve block was performed under ultrasound guidance,it is not only indicative of less pain and discomfort caused by the patients' tension and anxiety but also the alleviation of pain and discomfort caused by postural changes, thus confirming its value in postoperative adjuvant analgesia (10). Composite nerve block in the perioperative period of hip replacement surgery can reduce hospital costs, shorten hospital stays, and reduce the incidence of postoperative delirium and other complications (11).

The results of the present study suggest that nerve block is a protective factor for three-year mortality in patients. First, a nerve block is more effective than intravenous analgesia with opioids and reduces the risk of systemic complications (12). Hip fracture patients who received nerve blocks had lower pain scores, needed fewer pre- and postoperative opioids, and had lower postoperative pain scores than patients who received conventional pain management, thereby promoting early recovery. Additional peripheral surgical nerve blocks may reduce the incidence of postoperative delirium (13, 14). Although delirium is associated with an increased risk of postoperative mortality, patients with postoperative delirium had 30-day and 1-year mortality rates of 8.7% and 34.2%, respectively (15). Additionally, nerve blocks may reduce the incidence of postoperative mortality in patients by reducing the incidence of delirium.

General anesthesia before matching was found to be a protective factor for 3-year mortality in hip arthroplasty patients, but the difference after matching was not significant. The main reasons for this may be that this study was retrospective, that the choice of anesthesia could not be randomized, that general anesthesia tended to be chosen for patients in a better general condition and that intraspinal anesthesia tended to be chosen for patients with more comorbidities. Thus, after propensity score matching, the anesthesia modality did not affect the 3-year mortality rate of patients.

A small amount of crystalloid fluid infusion before matching was a risk factor for 3-year mortality in patients; however, the difference was not significant after matching. The reason for this is that in clinical work, we would perform goal-directed volume therapy according to the patient's condition, and anesthesiologists tend to restrict fluid infusion in patients with poor cardiac function because of their high risk of postoperative mortality. After matching, in patients with similar general conditions, intraoperative crystalloid fluid infusion did not affect the 3-year mortality rate.

Previous studies suggested that ASA grade, sex, age, and preoperative waiting time were risk factors for short- and long-term postoperative mortality; however, these indicators did not affect the incidence of postoperative mortality in our study. The reasons for this finding were mainly that the study was a single-center, retrospective study with a relatively small sample size, which may have biased the results, this is also a limitation of this study,a multicenter, prospective, large-sample study is needed to further validate the results in the future to provide guidance for the perioperative management of patients undergoing hip surgery.

In summary, nerve block is a protective factor for 3-year mortality, and composite nerve block with general anesthesia and neuraxial anesthesia deserves great promotion.

## Data Availability

The original contributions presented in the study are included in the article/Supplementary Material, further inquiries can be directed to the corresponding author.
